# Selective Factors Associated with the Evolution of Codon Usage in Natural Populations of Arboviruses

**DOI:** 10.1371/journal.pone.0159943

**Published:** 2016-07-25

**Authors:** Lauro Velazquez-Salinas, Selene Zarate, Michael Eschbaumer, Francisco Pereira Lobo, Douglas P. Gladue, Jonathan Arzt, Isabel S. Novella, Luis L. Rodriguez

**Affiliations:** 1 Foreign Animal Disease Research Unit, USDA/ARS Plum Island Animal Disease Center, Orient Point, New York, United States of America; 2 Oak Ridge Institute for Science and Education (ORISE), Oak Ridge, Tennessee, United States of America; 3 Autonomous University of Mexico City, Genomics Sciences Program, Mexico City, Mexico; 4 Laboratório Multiusuário de Bioinformática, Embrapa Informática Agropecuária, Empresa Brasileira de Pesquisa Agropecuária (Embrapa) Campinas, Brazil; 5 Department of Medical Microbiology and Immunology, College of Medicine and Life Sciences, The University of Toledo, Toledo, Ohio, United States of America; Virginia Tech, UNITED STATES

## Abstract

Arboviruses (arthropod borne viruses) have life cycles that include both vertebrate and invertebrate hosts with substantial differences in vector and host specificity between different viruses. Most arboviruses utilize RNA for their genetic material and are completely dependent on host tRNAs for their translation, suggesting that virus codon usage could be a target for selection. In the current study we analyzed the relative synonymous codon usage (RSCU) patterns of 26 arboviruses together with 25 vectors and hosts, including 8 vertebrates and 17 invertebrates. We used hierarchical cluster analysis (HCA) and principal component analysis (PCA) to identify trends in codon usage. HCA demonstrated that the RSCU of arboviruses reflects that of their natural hosts, but not that of dead-end hosts. Of the two major components identified by PCA, the first accounted for 62.1% of the total variance, and among the 59 codons analyzed in this study, the leucine codon CTG had the highest correlation with the first principal component, however isoleucine had the highest correlation during amino acid analysis. Nucleotide and dinucleotide composition were the variables that explained most of the total codon usage variance. The results suggest that the main factors driving the evolution of codon usage in arboviruses is based on the nucleotide and dinucleotide composition present in the host. Comparing codon usage of arboviruses and potential vector hosts can help identifying potential vectors for emerging arboviruses.

## Introduction

Rather than a taxonomic classification, arboviruses are a broad group of viruses transmitted biologically by hematophagous (blood-feeding) arthropod vectors (e.g. mosquitoes, ticks, biting flies) to vertebrate hosts [1]. This type of transmission cycle involves virus-host interactions with both invertebrate and vertebrate hosts. Arboviruses not only affect humans but also other animal species and some are transmitted and many cause disease in both humans and animals (zoonotic diseases). Understanding this three-component transmission cycle, is of great importance as recently arboviral infections have been seen with increasing frequency and magnitude for both old and newly emerging arboviruses (e.g. Zika virus). For example, dengue virus affects over 400 million people a year (http://www.cdc.gov/dengue/). Recent examples of emerging arboviral activity in human populations include the 2003 West Nile fever outbreak[2], and the Chikungunya virus, which reached the Western hemisphere sparking over 1.5 million new clinical cases [3]. A recent non-human arboviral infection; Schmallenburg virus was identified in Germany in 2011 has induced thousands of cases in eight European countries [4].

Arboviruses consist mostly of RNA virus with the exception of African swine fever virus a dsDNA virus[1], the RNA viruses that are classified as arboviruses are comprised of many different taxa, having in common only that they are viruses that infect vertebrate hosts but are transmitted by arthropod vectors. Current biological information regarding the infectious cycles of arboviruses shows substantial differences in vectors, hosts and transmission modes. For example, West Nile virus (*Flaviviridae*) is maintained and amplified in nature within an enzootic transmission cycle among birds and *Culex* mosquitoes, with outbreaks caused by tangential or spillover transmission to equids and humans, which may develop terminal neuroinvasive disease. However, these are considered dead-end hosts because they do not develop high enough viremia adequate for mosquito infection [1]. In some cases, the role of the vertebrate in the life cycle of the virus may be minimal. For example, many phleboviruses seem to be maintained in their vectors, phlebotomine sandflies, by vertical (transovarial) transmission, which may allow persistence during periods when susceptible vertebrate hosts are not available [5].

Viruses are intracellular pathogens that have to exploit and co-evolve with host molecular mechanisms to prosper in a cellular environment [6]. Most amino acids are encoded by 2–6 different synonymous codons due to the redundancy of the genetic code. Codon usage bias refers to the phenomenon that some synonymous codons are used more often than others and how this preference varies within and among species [7]. There are two non-mutually exclusive models that propose mechanisms to account for codon usage bias in viruses. The transitional model assumes that codon usage is under selection because RNA viruses are completely dependent of host tRNAs [8] and the bias results from viruses matching the codon usage of their hosts [9]. In addition, other factors such as mononucleotide and dinucleotide composition are likely to influence codon usage in RNA viruses. The second model proposes that mutational pressures and the probability of fixation for different mutations determine codon usage bias [10]. Evolution can sometimes favor viruses that match host codon usage to promote speed of replication, as is the case of poliovirus [11, 12, 13] and influenza A virus [14].

In order to investigate possible patterns of coevolution between arboviruses and their vertebrate and invertebrate hosts, we analyzed the base composition and codon usage bias of different arboviruses and their respective vertebrate and invertebrate host. Our results suggest that codon usage patterns among different arbovirus are consistent with the codon usage of their respective natural hosts, with the mimicking of nucleotide and dinucleotide compositions being the main factors that explain these patterns. Correlations between these factors may have practical application for the characterization of emerging arboviruses and the identification of their hosts and vectors.

## Materials and Methods

### Viral Dataset

**Fig 1:**
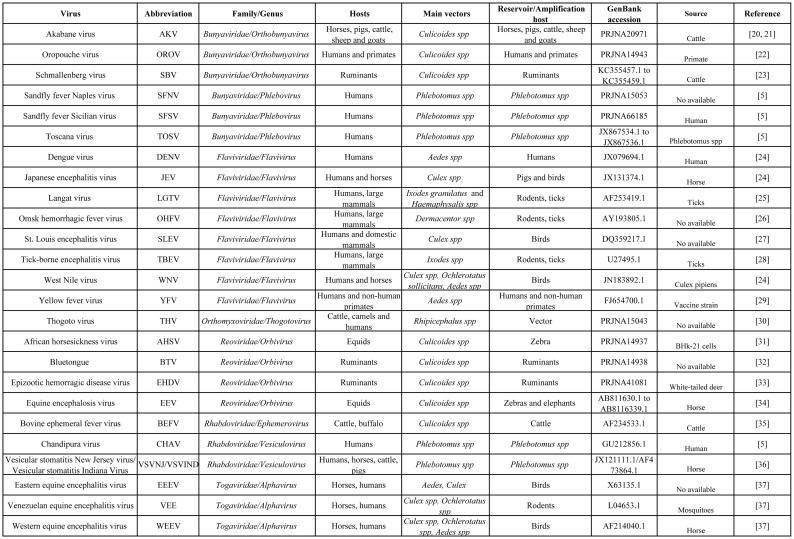
General information about viral species used in this study. Twenty-six different viruses comprised of 6 different viral families that represent the most common fully sequenced arboviruses were used for this study.

All complete viral genome sequences included in this study were downloaded from the National Center for Biotechnology Information [15]. These viral genomes were randomly chosen in order to obtain one representative member from each of the different viral families included in this study. Detailed information about the viruses used in this study is presented in Fig 1.

### Host Dataset

The host and vector codon usage and GC composition in coding regions were obtained from the Codon Usage Data Base [16], which includes sequences of complete protein-coding genes. The vertebrate dataset was composed of the following species: Cow (*Bos taurus*), horse (*Equus caballus)*, chicken (*Gallus gallus*), human *(Homo sapiens)*, turkey (*Meleagris gallopavo)*, mouse (*Mus musculus*), chimpanzee (*Pan troglodytes*) and pig (*Sus scrofa)*. The invertebrate dataset included insects and arachnids: mosquitoes in the family *Culicidae* (*Aedes aegypti*, *Aedes albopictus*, *Culex nigripalpus*, *Culex pipens quinquefasciatus*, *Culex pipiens*, *Culex tritaeniorhynchus* and *Ochlerotatus sollicitans)*, sand flies in the family *Psychodidae* (*Lutzomyia longipalpis*, *Phlebotomus argentipes*, *Phlebotomus ariasi*, *Phlebotomus duboscqi*, *Phlebotomus papatasi*, *Phlebotomus perniciosus*), midges in the family *Ceratopogonidae (Culicoides sonorensis)*, as well as the tick species *Ixodes rinicus* and *Rhipicephalus microplus* of the family *Ixodidae*.

### Compositional bias measures

For each virus included in this study, codon usage, overall GC-content, and the GC_1_, GC_2_, and GC_3_ (GC content at the first, second and third codon position, respectively) were calculated using the CAIcal software [17].

Dinucleotide odds ratio is defined as the quotient of the probability of finding a dinucleotide in a given sequence divided by the product of the probabilities of finding each nucleotide that forms the pair in the same sequence, calculated as shown in the following equation: P_xy_ = (*f*_xy_)/(*f*_x_*f*_y_). Where *f*_x_ and *f*_y_ denote the frequency of mononucleotides *x* and *y* in a given sequence and *f*_xy_ denotes the frequency of dinucleotide *xy* in the same sequence. In the case of organisms with double-stranded genomic material, the frequency of each dinucleotide must be calculated in a symmetric manner, also considering the complementary strand as described in the following equation: P_xy_ = 2(*f*_xy_ +*f*_zw_) / (*f*_x_ +*f*_y_) (*f*_z_ + *f*_w_). Where *x* and *y* denote two dinucleotides, and *z* and *w* denote the two complementary nucleotides of *y* and *x*, respectively [18].

Dinucleotide odds ratios for all viral sequences used in this study were calculated using single-stranded odds ratios; the only exception being viruses from the *Reoviridae* family, which contain a double RNA strain in their genome. In this case, we used the same method applied to the hosts and calculated the dinucleotide odds ratio using the symmetric odds ratio.

### Amino acid composition calculation

Average amino acid compositions among different host proteins were obtained directly from the Codon Usage Data Base, which uses coding sequence composition for to determine Codon Usage for individual species. For the viruses, the amino acid composition was inferred considering the protein coding region for the viruses under study. Calculations were conducted in the ExPASy Bioinformatics Resource Portal using the software ProtoParm [16].

### Relative Synonymous Codon Usage (RSCU)

RSCU is a common measure used to estimate codon bias for all codons that code for any amino acid with a degeneracy greater than one (i.e. all except methionine and tryptophan). It is defined as the observed frequency of the codon *j* in a sequence *x* divided by the expected frequency *E* if all synonymous codons for the amino acid coded by *j* were equally frequent. Calculations were conducted using the following equation: RSCU*j*(x) = (*f*_*j*_^x^ /E_*j*_^*x*^,) Where *f*_j_^x^ is the observed frequency of codon *j* in the genome *x* and *E*_*j*_^*x*^ is the expected frequency of the codon *j*. Expected values are calculated by counting the total number of synonymous codons for a given amino acid in the sequence divided by the number of existing codons that code for it. RSCU values larger than 1.0 indicate that a given synonymous codon is favored over the rest; RSCU values less than 1.0 indicate a disfavored codon; and RSCU values of 1.0 indicate no preference [19].

### Two-way hierarchical clustering analysis (TWHCA)

Two-way hierarchical clustering analysis is a statistical method that classifies objects into groups (clusters) according to similarities between them, and is used to identify a subset in one dimension that is useful for clustering the other dimension [20]. We organized all organisms (viruses, hosts and vectors), in a matrix of *N* x *M* dimensions, with *N* being the number of species and *M* the number of degenerate codons, represented by their RSCU values. Monocodonic amino acids (tryptophan and methionine) and stop codons (UAA, UAG and UGA) were excluded to generate a final multivariate data set of 59 codons for each organism. The original dataset with all RSCU values is included in S1 Table. For the cluster analysis, the values for each codon were scaled and centered by subtracting the mean and dividing by the standard deviation. With the standardized values, cluster analysis using Ward’s minimum variance method [21] in combination with initial Manhattan (city-block) distance measures was conducted in the R statistical environment. Ward’s method joins clusters based on minimizing the within-group sum of squares and tends to produce compact clusters. The results of the cluster analysis are presented as dendrograms, and the order of clusters is used to reorder the rows and columns of a heat map showing the RSCU values.

To assess the uncertainty in the first dimension of the hierarchical cluster analysis, approximately unbiased (AU) p-values were calculated by a multiscale bootstrap procedure [22]. The AU values are expressed as percentages and are superimposed over the corresponding dendrograms. For a cluster with a given AU p-value, the null hypothesis of non-existence of the cluster is rejected at a significance level of (100-AU)/100; i.e., for high AU values, it can be assumed that these clusters do actually exist in the original data, and are not merely caused by sampling error.

### Principal component analysis (PCA)

PCA is an orthogonal linear transformation that converts the original data set into a new coordinate system, in which the greatest variance represented by any projection of the data (the first principal component; PC) comes to lie on the first coordinate and the second greatest variance on the second PC [23]. This method is frequently used to analyze multivariate data sets. Reliable components in the analysis where retained based on the eigenvalues (the variance of the principal components), applying the criteria of the eigenvalues greater than one rule [24].

Correlations between different variables and the main principal components were examined by correlation analysis and analysis of variance (ANOVA) using the statistical software JMP 11.

## Results

### Hosts are influencing codon usage in arboviruses

**Fig 2:**
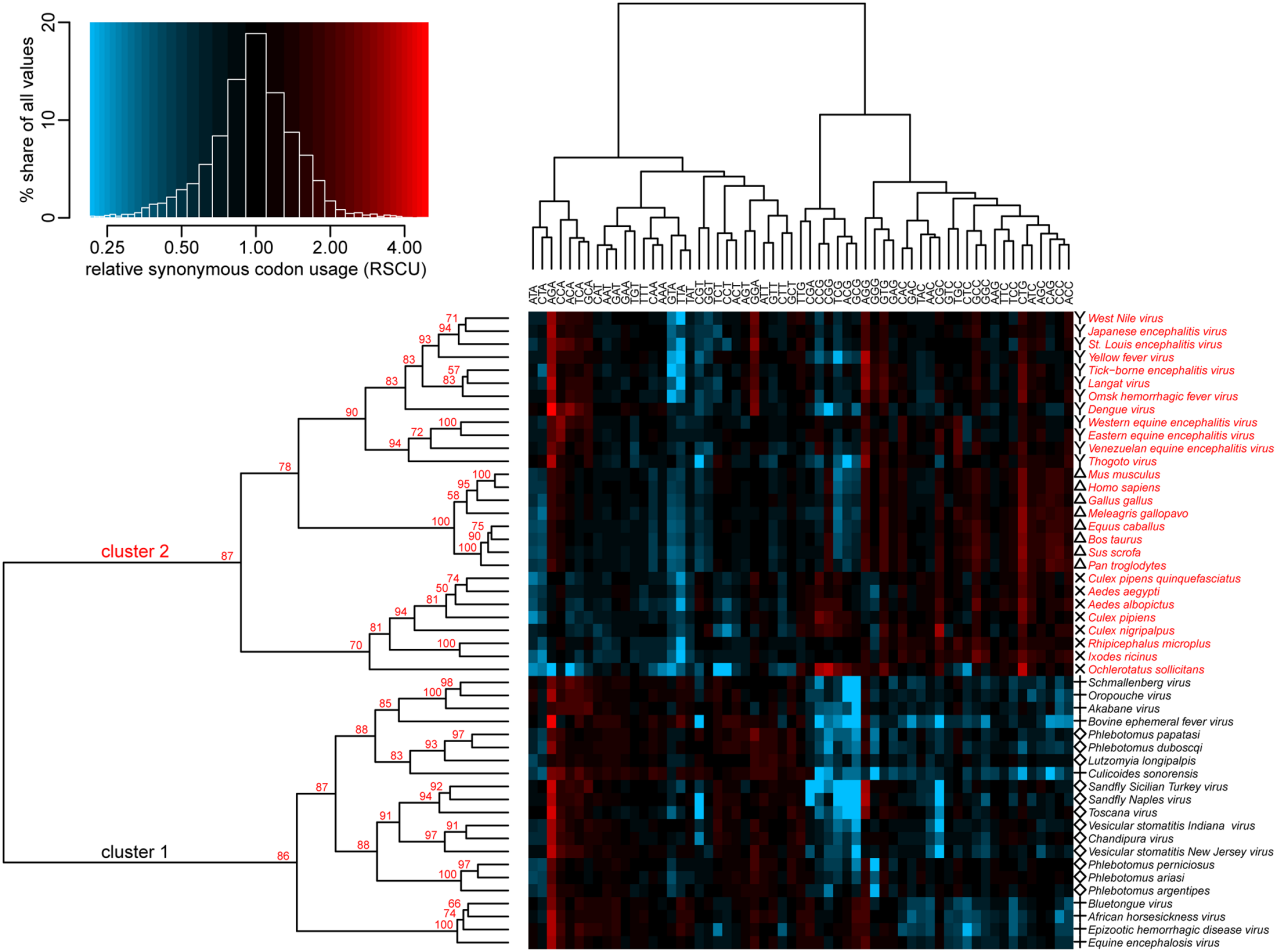
Influence of host on codon usage in arboviruses. The two-way hierarchical cluster analysis shows a correlation of the RSCU between viruses and their respective hosts. In the first dimension, viral and invertebrate species were split into two main clusters, while vertebrate species clustered together, in the second dimension, there was a contrasting pattern between species based on their preference for A/T or G/C-end codons. The cluster analysis was done with centered and scaled RSCU values, but cell colors in the heat map represent the original values. High RSCU values are shown in red and low values in blue; values around 1 are shown in black. The AU p-values from a multiscale bootstrap analysis (n = 10000) are overlaid over the first-dimension dendrogram.

TWHCA was utilized to investigate intra-species differences in codon usage and evaluate similarities among species. In the first dimension of the analysis, there was a clear division of all species, viral and eukaryotic, into two main clusters labeled as cluster one and cluster two (Fig 2). This cluster division was further supported by the multivariate bootstrap analysis, in which unbiased p-values (AU) of 88 and 86 were obtained for cluster one and two, respectively.

In the first dimension of these analyses, cluster one included all viruses from the genera *Vesiculovirus*, *Ephemerovirus*, *Orthobunyavirus*, *Phlebovirus* and *Orbivirus*, together with their main invertebrate vectors, which belong to the families *Psychodidae* and *Ceratopogonidae*. Within cluster one, viruses associated more closely with their respective vectors than with other related viruses. For example, the *Culicoides*-transmitted viruses, particularly AKV, OROV, SBV and BEFV, grouped closely with *Culicoides sonorensis*, while SFNV, SFSV, TOSV, VSNJV, and VSIV grouped closely with members of the *Psychodidae* family (their associated sand fly vectors).

Cluster two included the genera *Alphavirus*, *Thogotovirus* and *Flavivirus*, which were similar in their codon usage to both vertebrates (mammals and birds) and invertebrates of the families *Culicidae* and *Ixodidae*. Unlike the species in cluster one, the distinctive codon usage patterns of species in cluster two placed them into subgroups retracing their phylogenetic origin rather than the biological interaction between viral species and their respective vector or host.

### Patterns of codon usage between arboviruses

Analyzing the second dimension of the TWHCA, it is possible to distinguish two main clusters based on their preference for codons with A/T or G/C endings. Although the viruses were split into two different clusters, all of them shared a common pattern of codon usage correlated with the usage of codons TCA, AGA, ACA, GCA, AGG, TTG and CCA, for which the average RSCU for all viral groups was higher than one. With the exception of the orbiviruses, the remaining viral groups avoided the usage of certain codons containing CpG dinucleotides, such as TCG, CCG, ACG, GCG and CGA, where the average RSCU was lower than one. The same pattern of low usage of codons containing CpG was displayed only by the vertebrate group and the invertebrates associated with cluster one. Invertebrates associated with cluster two had a high preference for the usage of these codons.

A second pattern of usage reflected the preferences among viruses and their respective hosts. In the case of viruses associated with cluster one, similar preferences of usage between them and their hosts were seen for codons TTT, TTA, TAT, ATA, CAT, AAT, GAT, GGT, AAT, TCT, CCT, ACT and GCT. For viruses in cluster two, these similarities were seen for codons CTG, ACC, ATC, AGC and CAC.

**Fig 3:**
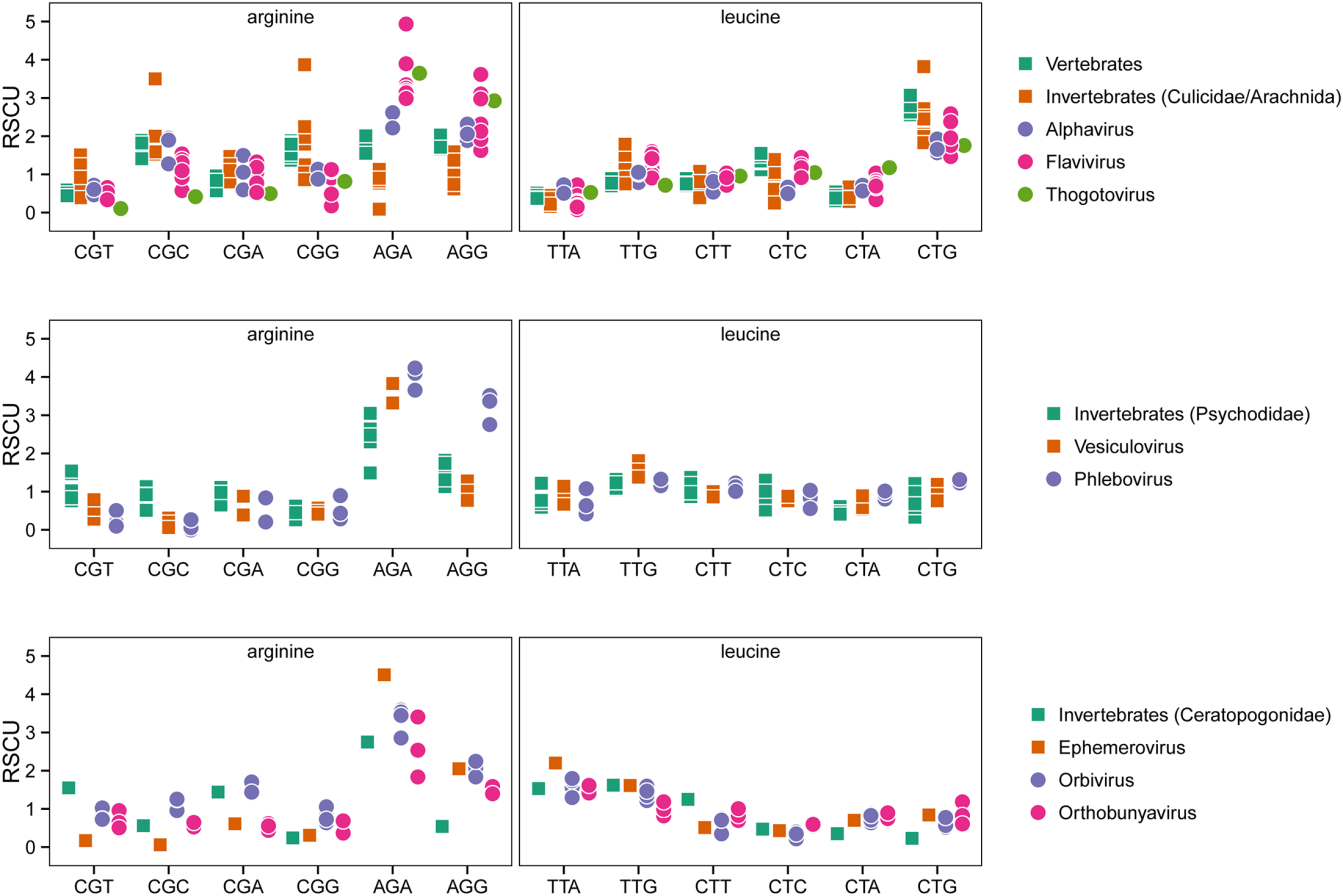
Frequencies of relative synonymous codon usage for encoding the amino acids leucine and arginine. RSCU for the different codons to encode the amino acids leucine and arginine, were calculated and compared between different groups of viruses and their respective hosts and for each virus and host. Each dot in the graphic represents a single virus or host. Different families of hosts and viruses were represented in different colors. In the case of leucine, Flaviviruses, alphaviruses and thogotoviruses displayed the same high predilection for the CTG codon as their natural hosts, using this codon as a first option to encode leucine, while in case of arginine, AGA was the codon with the highest RSCU among viral populations independently of their host preferences.

Interestingly, the RSCU values of all 59 codons analyzed in this study were fairly disparate across the two main clusters. Based on their RSCU values, two of the most relevant codons identified by this analysis were CTG and AGA. CTG, one of the six codons used to encode leucine, had the highest RSCU difference between the two main clusters (1.54±0.45), with average values of 0.85±0.34 for species contained in cluster one and 2.39±0.57 for species contained in cluster two. Among the six available codons for the amino acid leucine, the CTG codon had the highest RSCU within the coding regions analyzed in vertebrates (2.82±0.19) and invertebrates of the *Culicidae* family (2.67±0.62). Flaviviruses, alphaviruses and thogotovirus displayed the same predilection for the CTG codon, contrasting with viruses in the genera *Vesiculovirus* and *Phlebovirus* usually associated with invertebrates of the *Psychodidae* family, as well as with ephemeroviruses, orbiviruses and orthobunyaviruses commonly associated with invertebrates of the *Ceratopogonide* family (Fig 3). Contrastingly, AGA, one of the six codons used to encode arginine was by far the codon with the highest RSCU (3.33 ±0.77) among viral populations, independently of their host preference, suggesting that a common evolutionary pattern among may be favoring the high usage of this codon.

### Factors influencing codon usage in arboviruses

**Fig 4:**
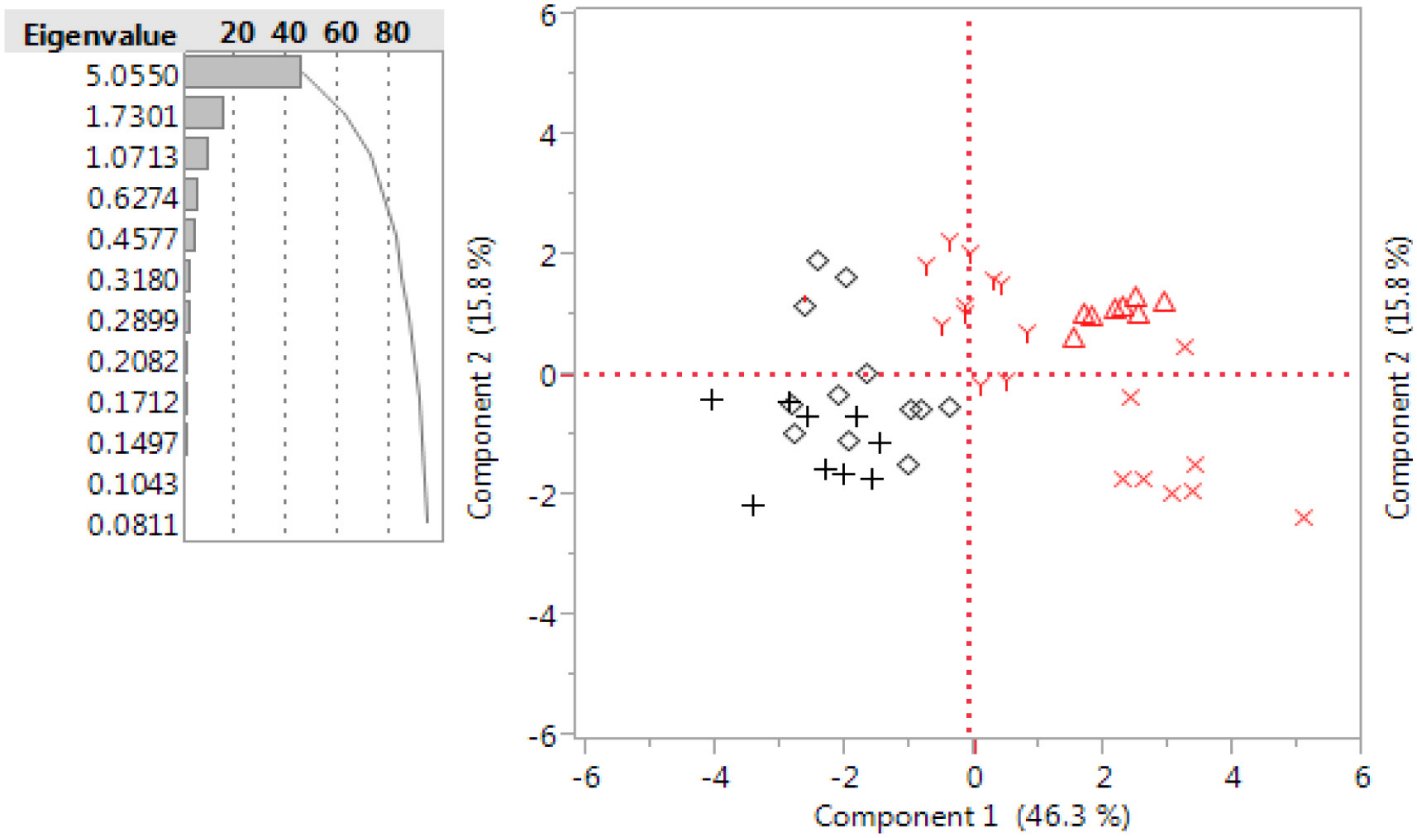
Two main principal components explain 62.1% of the total variation among RSCU indices. The principal component analysis was conducted using RSCU values corresponding to 59 codons of all eukaryotic and viral species used in this study. Based on eigenvalues of 5.05 and 1.73 the first principal component one accounts for the 46.3% of the total variance, while the second principal component for the 15.8%. In black are represented the following species: (+) *Orthobunyavirus*, *Ephemerovirus*, *Orbivirus* and *Ceratopogonide*. (◊) *Vesiculovirus*, *Phlebovirus* and *Psychodidae*. In red are represented the following species: (Y) *Flavivirus*, *Alphavirus* and *Thogotoviridae*. (Δ) Vertebrates. (X) *Culicidae* and *Arachnida*.

In order to gain a more thorough understanding of which factors, might be relevant in the choice of codon usage in arboviruses and their respective vectors and hosts, we conducted principal component analysis (PCA). The results showed that the two principal components were able to explain 62.1% of the total variation among the 59 RSCU indices. The first principal component with an eigenvalue of 5.05 accounted for 46.3% of the total variance, while the second principal component with an eigenvalue of 1.73 contributed with 15.8% of the differences. Viral species were clustered in two separate groups, each containing their respective vectors or hosts, confirming the prior observations in the TWHCA and providing further support to the hypothesis that host or vector codon usage are major determinants of virus codon usage (Fig 4).

### Relevant codons associated with the two principal components

Correlation analysis was utilized in order to identify which codons correlated best with the two first principal components. There were significant positive and negative correlations (p<0.05) between the first principal component and several codons, associated with the two main clusters of the TWHCA (Fig 3. The highest positive correlation was with CTG (r = 0.94), followed by ACC (r = 0.87), GGC (r = 0.81), CAC (r = 0.81), CAG (r = 0.77), CGG (r = 0.77), GTG (r = 0.77) and negative correlations were found for AAA (r = -0.9), AAT (r = -0.87), ATT (r = -0.86), CAA (r = -0.84), TTA (r = -0.82), TAT (r = -0.82) and TCA (r = -0.78). In addition, we found significant negative and positive correlations (p<0.05) with the second principal component for codons TCG (r = 0.75), CCG (r = 0.66), ACG (r = 0.52), TTC (r = 0.52), GAT (r = 0.46), CGT (r = 0.44), AGG (r = -0.66), AGA (r = -0.64), ACA (r = -0.63), GGG (r = -0.62), GCA (r = -0.55) CTA (r = -0.45).

### Nucleotide and dinucleotide compositions are the main factors influencing codon usage in arbovirus

#### Nucleotide composition

To determine the influence of nucleotide, dinucleotide and amino acid compositions on the variability of codon usage, these parameters were calculated for each species and ANOVA was used to examine correlation with the two main principal components. The results indicated that differences in GC nucleotide composition were by far the best correlated variable with the first principal component. Significant associations (p<0.0001) were found for GC_3_ (R^2^ = 0.87), GC_2_ (R^2^ = 0.74) and GC_1_ (R^2^ = 0.61), suggesting that GC_3_ is the main factor influencing the variability associated with the first principal component. In general, vertebrates, invertebrates in the family *Culicidae* or class *Arachnida*, as well as viruses from the genera *Alphavirus*, *Flavivirus* and *Thogotovirus* have the highest total percentage of GC_3_, accounting for more than 50% of the total mononucleotide content. In contrast, invertebrates in the families *Psychodidae* and *Ceratopogonidae* and viruses in the genera *Vesiculovirus*, *Ephemerovirus*, *Orthobunyavirus*, *Phlebovirus* and *Orbivirus* have a total GC_3_ content below 50%. These differences correlate strongly (p<0.0001) (R^2^ = 0.85) with the usage of the CTG codon among species in this study.

#### Dinucleotide composition

**Fig 5:**
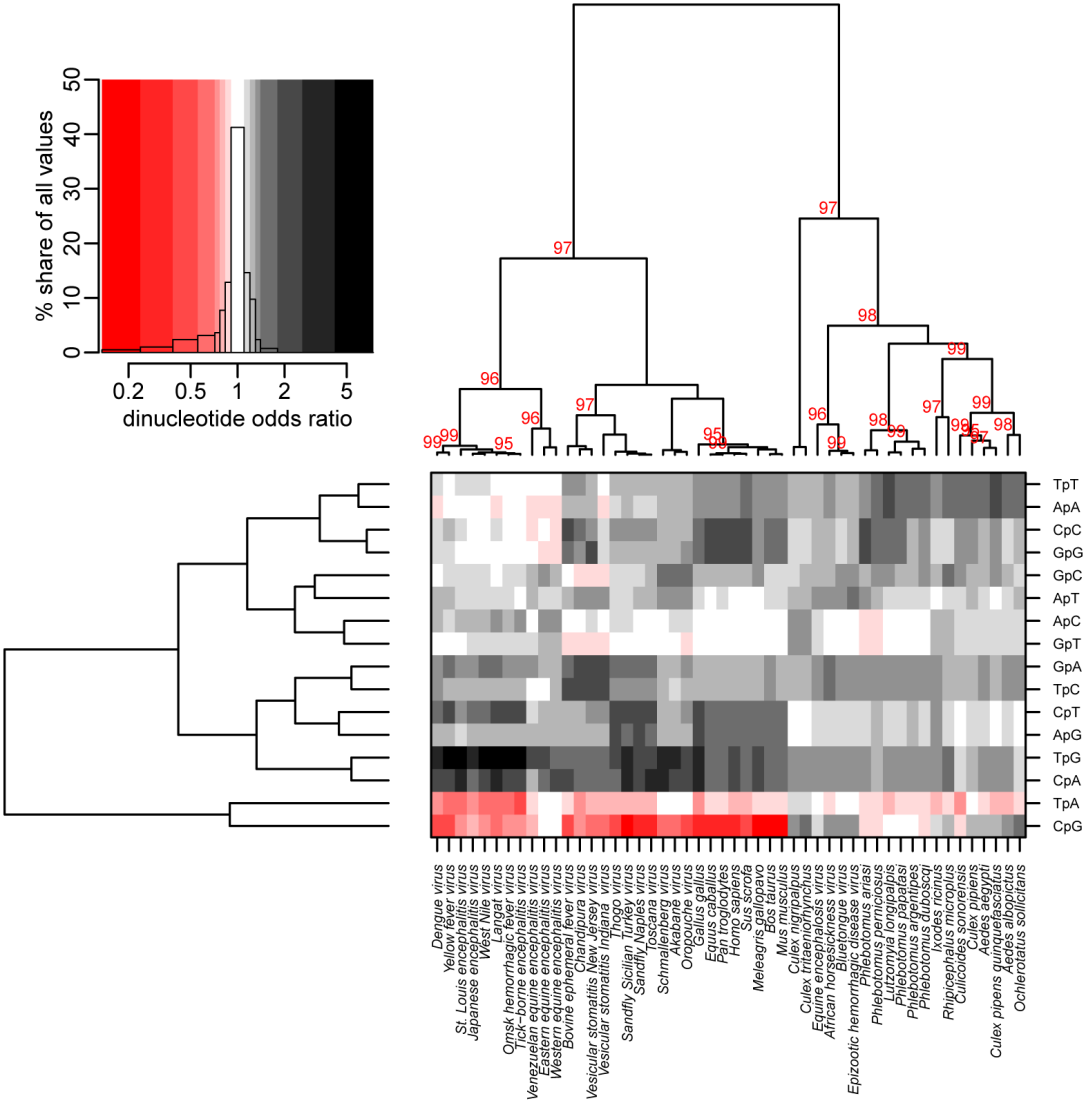
Dinucleotide odds ratios in most arboviruses resemble the vertebrate hosts. Hierarchical cluster analysis was conducted using the dinucleotide composition of all species included in this study. AU values of 97 (n = 10000) support the existence of two main clusters with either invertebrates or vertebrates. With the exception of orbiviruses, which clustered with invertebrates, the arboviruses grouped together with the vertebrates.

Dinucleotide composition was the variable that was most significantly associated with the second principal component. There were significant correlations (p<0.0001) with dinucleotides CpT (R^2^ = 0.87), ApG (R^2^ = 0.64), CpG (R^2^ = 0.63), CpA (R^2^ = 0.66) and TpG (R^2^ = 0.65). Comparing patterns of dinucleotide usage among viruses, vertebrates and invertebrates hosts, using TWHCA, we found that the hosts were assigned to separate clusters. With the exception of orbiviruses, which clustered with invertebrates, the arboviruses grouped together with the vertebrate group and presented a typical dinucleotide pattern with significant underrepresentation of dinucleotides CpG and TpA and overrepresentation of dinucleotides CpA, and TpG, (Fig 5). These results suggest that viruses acquire these dinucleotide patterns as a consequence of their replication in the vertebrate host and these patterns shape codon usage similarities between viruses associated with different clusters (cluster one vs. cluster two) in the TWHCA based on RSCU values.

### Amino acid composition

Comparing amino acid frequencies by the Tukey-Kramer test, leucine (Leu) with an average of 9±1% (p<0.05) was predominant among the proteins of both viruses and hosts (S1 Fig). However, isoleucine (Ile) had the best association with the first principal component (p<0.0001, R^2^ = 0.53). Differences in Ile composition correlated best with the general CG composition (p<0.0001, R^2^ = 0.73). Interestingly, it has been demonstrated previously that a strong bias in the nucleotide composition can also affect the average amino acid composition of the encoded proteins, suggesting that AT-rich coding sequences would encode proteins with excess of FYMINK amino acids (phenylalanine, tyrosine, methionine, isoleucine, asparagine, and lysine), whereas GC-rich sequences would produce proteins with high levels of GARP amino acids (glycine, alanine, arginine, and proline) [25]. The influence of GC content on amino acid composition was probed using ANOVA to look for associations between GC content and the GARP/FYMINK ratio. The results demonstrated a statistically significant linear correlation (p < 0.0001) between that ratio and GC (R^2^ = 0.61), GC_1_ (R^2^ = 0.67), GC_2_ (R^2^ = 0.77) and GC_3_ (R^2^ = 0.38). Thus, GC composition is not only influencing 46.2% of the RSCU variance but also might be influencing the differences in amino acid composition between different species. However, because our analysis was only done using the coding region of arboviruses, we cannot rule out the possibility that amino acid composition is driving base composition.

### Biological markers to infer host preferences

**Fig 6:**
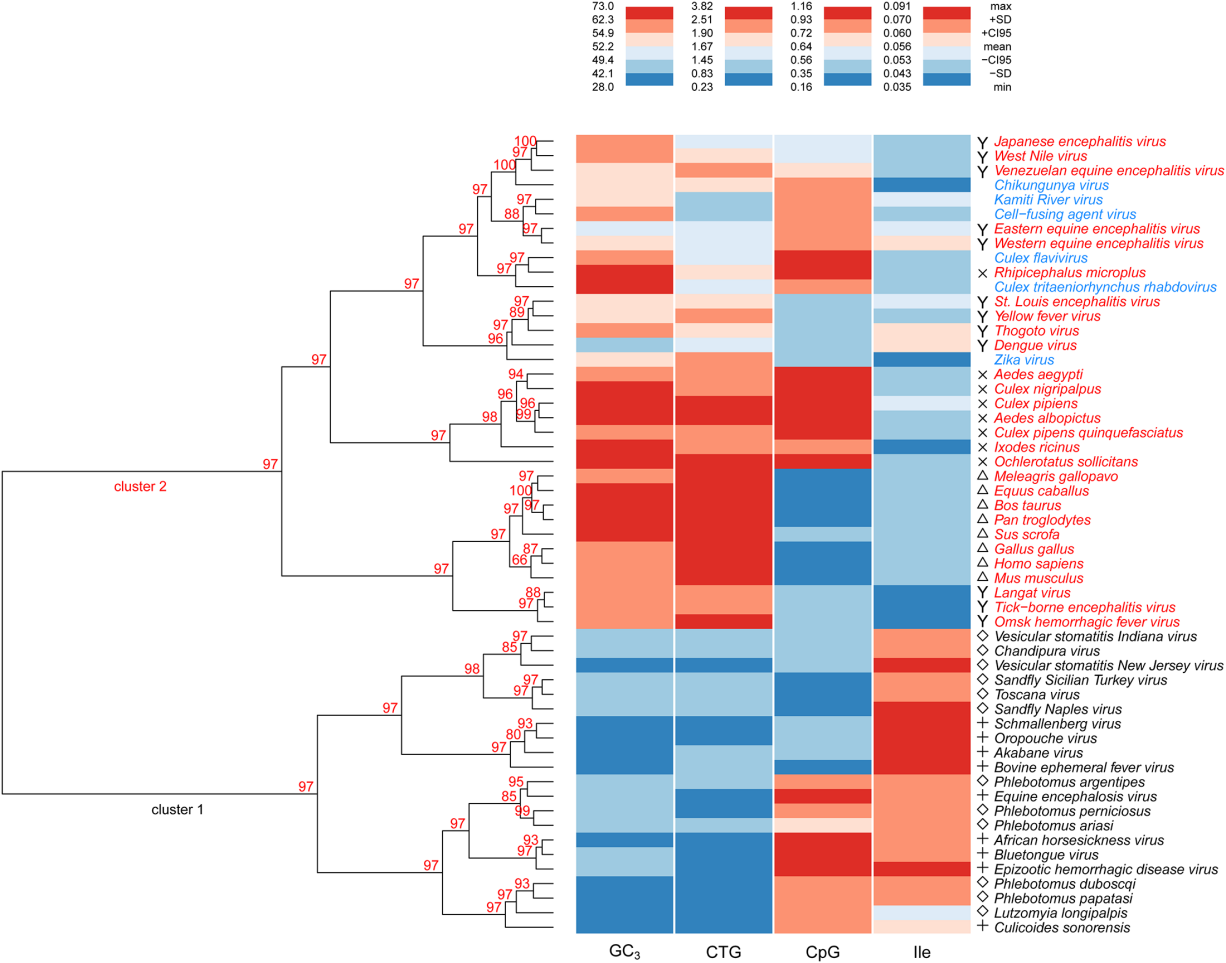
Inferring arbovirus host preferences. A two way hierarchical cluster analysis (Euclidean distance, Ward’s D clustering) was conducted using some of the relevant markers identified in this study (GC_3_, CTG, CpG, Ile). The cluster relationship was congruent with the current biological information regarding to the different infectious cycles of these viruses. The AU p-values from a multiscale bootstrap analysis (n = 10000) are overlaid over the dendrogram Viruses with blue labels represent new viruses included to assess the reliability of this analysis.

Based on the differences in codon usage, nucleotide, dinucleotide and amino acid compositions among different arboviruses and their vertebrates and invertebrates hosts, it is possible to propose relevant biological markers that might be useful for the characterization of future emerging arboviruses. For this propose, using the values of GC_3_, CTG, CpG, and Ile, it was conducted a new TWHCA in order to confirm the previous cluster association obtained using RSCU values. Additionally, the potential use of this analysis was demonstrated adding six new viruses. Four viruses were chosen based on their ability to replicate solely in invertebrate cell culture and were isolated from insect cell lines or from field-collected mosquitoes. Specifically viruses in this category and their sources were: cell-fusing agent virus (flavivirus), isolated form *Aedes aegypti* [26], Kamiti River virus (flavivirus), isolated from *Aedes macintoshi* [27], Culex flavivirus (flavivirus) isolated from *Cluex pipiens* [28] and *Culex tritaeniorhynchus* rhabdovirus isolated from *Culex tritaeniorhynchus* [29]. Additionally, two different reemerging arboviruses were chosen: Chikungunya virus (Alphavirus) and Zika virus, both transmitted by Aedes mosquitoes [30,31]. Using just these four biological markers, a biologically-relevant cluster of viruses and their invertebrate host was obtained, statistically supported by high AU values (Fig 6). The only exceptions were LGTV, OHFV and TBEV (transmitted by ticks) that appear more associated with the vertebrate cluster. Interestingly, although phylogenetically *Culex tritaeniorhynchus* rhabdovirus, is more related to the rest of the rest of rhabdoviruses used in this study (BEFV, CHAV, VSVNJ and VSVIND), it grouped in the opposite cluster clearly influenced by its host preference, associated with viruses transmitted by mosquitoes of the *Culicidae* family.

## Discussion

In the investigation described here we sought to understand possible selective factors associated with the evolution of codon usage in natural populations of arboviruses. For this purpose we used a combination of different bioinformatic tools to analyze the complete genomes of 26 arboviral species from six different viral families.

### Vertebrates and invertebrates could influence codon usage in distinct arboviruses lineages

Subsequently we used HCA to test the hypothesis that specific virus-host interactions play a role in arboviral RSCUs. Among hosts, vertebrates displayed the lowest variation in codon usage bias and all species were found in a specific sub-cluster. In contrast there was substantial variation among the invertebrates, which appeared split into the two main clusters. These patterns are consistent with a previous study in which the vertebrates demonstrated lower variation of codon usage bias compared with the average of other groups of eukaryotes as well as prokaryotes [32]. The authors of that study suggested that there is a negative correlation between codon usage bias diversity and genomic complexity.

In contrast to vertebrates, insects are considered the most diverse organisms in the history of eukaryotic life, with a projected number of recognized species close to one million. [33]. The insect order Diptera (which includes *Culicidae*, *Psychodidae* and *Ceratopogonide* familes) is the most species-rich, anatomically varied and ecologically innovative group, making up 10–15% of known animal species [34]. A previous study using 22 insect species of order Diptera (*Culicidae* and *Drosophila*) and Hymenoptera showed the contrasting patterns of codon frequencies between these two orders [35]. In the current study, we have demonstrated that there is high codon usage diversity associated with the Diptera order. This is interesting, because the high diversity of codon usage identified in the invertebrate group was consistent with the pattern shown by arboviruses, which grouped into the two main clusters, suggesting that invertebrates rather than vertebrates might be responsible for differences in codon usage between arboviruses. This is consistent with the fact that the evolutionary origin of most of the viral families included in this study is within insects [36] However, it is possible that the differences in clustering between vertebrates and the two main clusters of invertebrates could be influenced by shared ancestry between the invertebrates in each cluster, and the clustering in the virus families could be due to a common ancestor within the viruses studied.

Although all viral species included in this study have been recovered from vertebrates in nature, invertebrates can be involved in completing the infectious cycle for these viruses in nature. For example, a study conducted in Barkehji-Senegal from 1990 to 1995, used insect surveillance that determined CHAV and WNV were only isolated from their biological vectors (phlebotomine sand flies and *Aedes* species, respectively) [37]. Because other invertebrates were commonly found in the same area, and no CHAV or WNV were found in other invertebrates, it suggests that the main phase of restriction of arboviral replication could be the invertebrate host. In VSV, Phlebotomine sand flies are the only vector in the absence of clinical cases, to be confirmed biologically to host VSV. However during epidemics VSV has been isolated from midges (*Culicoides* spp and black flies) and mosquitoes (*Aedes* spp),[38]. Different *in vivo* and *in vitro* experiments conducted using VSIV, DENV and VEEV have also suggested the preponderance of the insect phase over the mammalian phase in evolutionary terms, suggesting that this may be a common feature of arbovirus evolution [16,17,39,40].

### Codon usage could reflect the natural transmission cycle in arboviruses

Cluster analysis of codon usage appeared to be consistent with biological studies of host and vector competence during natural transmission cycles. For example, in viruses belonging to the *Alphavirus*, *Thogothovirus* and *Flavivirus* genera, codon usage bias appeared related to both the vertebrate and the invertebrate host, while for viruses in the *Vesiculovirus*, *Ephemerovirus*, *Orthobunyavirus*, *Phlebovirus* and *Orbivirus* genera, codon usage bias remained clearly distinct from that of vertebrates and clustered just with their invertebrate host. In the first group of viruses, vertebrates play an important role in maintenance of the virus in nature and vertebrate reservoirs produce transient, high-titer viremias that allow transmission of the virus to feeding mosquitoes or ticks. Additional vertebrates, like humans or horses, can be infected but are considered dead-end hosts because of their lack of ability to produce sufficient viremia. In some cases, amplifier hosts, like birds infected with SLEV or with WNV, may develop high viremia without producing any symptoms or adverse effects on their health, thus increasing the possibility of transmission [41,42]. Conversely, in the second group viruses, that were recovered from vertebrate infections in nature, codon usage appears different than the codon usage of vertebrates. Previous research done in phleboviruses and VSV has produced inconclusive results trying to identify a vertebrate reservoir for the long-term persistence suggesting the possibility that long-term persistence is maintained solely by their respective vector. [43–45]. Finally, experimental work carried out with BTV indicates that once the virus infects a *Culicoides* vector, the midge will be able to infect a vertebrate host between 10–14 days after infection, corresponding to the period of time between the ingestion and the replication of the virus in the salivary gland. [46].

### Matching nucleotide and dinucleotide host compositions could be a mechanism influencing codon usage in arboviruses

The principal component analysis was used in order to identify potential factors influencing codon usage bias in arboviruses. Our results were consistent with previous research conducted in flaviviruses that determined hosts ware an important factor in shaping nucleotide motifs in flaviviruses [18]. Our results suggest that differences in nucleotide and dinucleotide compositions also influenced patterns of codon usage in distinct arboviruses. Experimental evidence in human immunodeficiency virus (HIV) showed that the biased nucleotide composition of HIV RNA is detected in human cells and induces a stronger immune response as compared to HIV RNA that had human optimized codon usage bias[47]. Studies conducted in polioviruses have also shown that differences in the nucleotide composition even at silent sites can determine the mutational robustness, evolutionary capacity and virulence, all factors that facilitate replication and spread within the dynamic host environment [13].

In the case of dinucleotide composition, selection favoring CpG depletion in RNA viruses has been associated with decreasing the innate immune response imposed by the vertebrate host [48]. This response may be mediated by the intracellular Pattern Recognition Receptor (PRR) Toll-like receptor 9 (TLR9), which recognizes in vertebrates CpG unmethylated DNA as a sign of infection [49]. Some experimental work conducted in echovirus (*Picornaviridae* family), altering the frequencies of CpG dinucleotides in the viral composition of this virus, showed that those viruses with increased frequencies of CpG had impaired replication kinetics and higher physical particule/infectious particle ratios as well as higher expression of mRNA for tumor necrosis factor and interferon beta genes compared with the wild type virus. Interestingly, the mutants with CpG dinucleotide depletion, showed enhanced replication and outcompeted wild-type virus during co-infections [50]. This work suggested that some viruses could have evolved with decreased CpG to avoid detection with TLR9, and thus avoid recognition by the immune system.

### Relevance of the CTG codon

Based on the contrasting usage preferences between the species used in this study, CTG was one of the most relevant codons. In the case of vertebrates, previous research showed a correlation between tRNA abundance and CTG preference in 12 out of 24 species [51]. Two species (*Gallus gallus* and *Mus musculus)* were also included in the current study. Interestingly, the mammalian CTG codon has evolved as an alternative start codon, contributing to the production of protein isoforms [52], a fact that might explain the high RSCU values for this codon in vertebrates. Only two insect species are available in the Genomic tRNA Database [53], one associated with the *Culicidae* family (*Anopheles gambiae*) and the other with the *Drosophilidae* family (*Drosophila melanogaster*). Both of these species demonstrated the same correlation between tRNA composition and the preference for CTG codon. This suggests the possibility that transitional selection (abundance of tRNA in a cell) might be one of the forces associated with the usage of this codon. However currently with only these two insect species available to evaluate the tRNA composition and the preference for the CTG codon its possible that other insect species may not have the same preference towards the CTG codon.

Viral hosts have been demonstrated to be an important factor in shaping nucleotide composition, amino acid composition and codon usage, it is possible as more sequenced arboviruses become available, and the codon usage data for more insect species is determined, that new codon bias factors could be identified. The main factors identified in this study, are a first step, and have shown promise to predict the host and vector species associated with newly identified viruses with unknown natural transmission cycles. However further analysis will be required to identify other codon bias factors that could be used to rationally predict the hosts for emerging arboviruses.

## Supporting Information

S1 Fig Amino acid composition. Tukey-Kramer analysis conducted among vertebrate, invertebrate and virus groups to determinate the frequency of amino acids among the proteins analyzed in this study.
(TIF)

S1 Table Database containing RSCU, observed/expected dinucleotide odds ratios nucleotide and amino acid composition values, from all species used in this study. (XLSX)
