# Supplementary figures and images for: Selective Factors Associated with the Evolution of Codon Usage in Natural Populations of Arboviruses
Source: PLoS One. 2016 Jul 25;11(7):e0159943. doi: 10.1371/journal.pone.0159943 (PMC4959722; doi:10.1371/journal.pone.0159943)

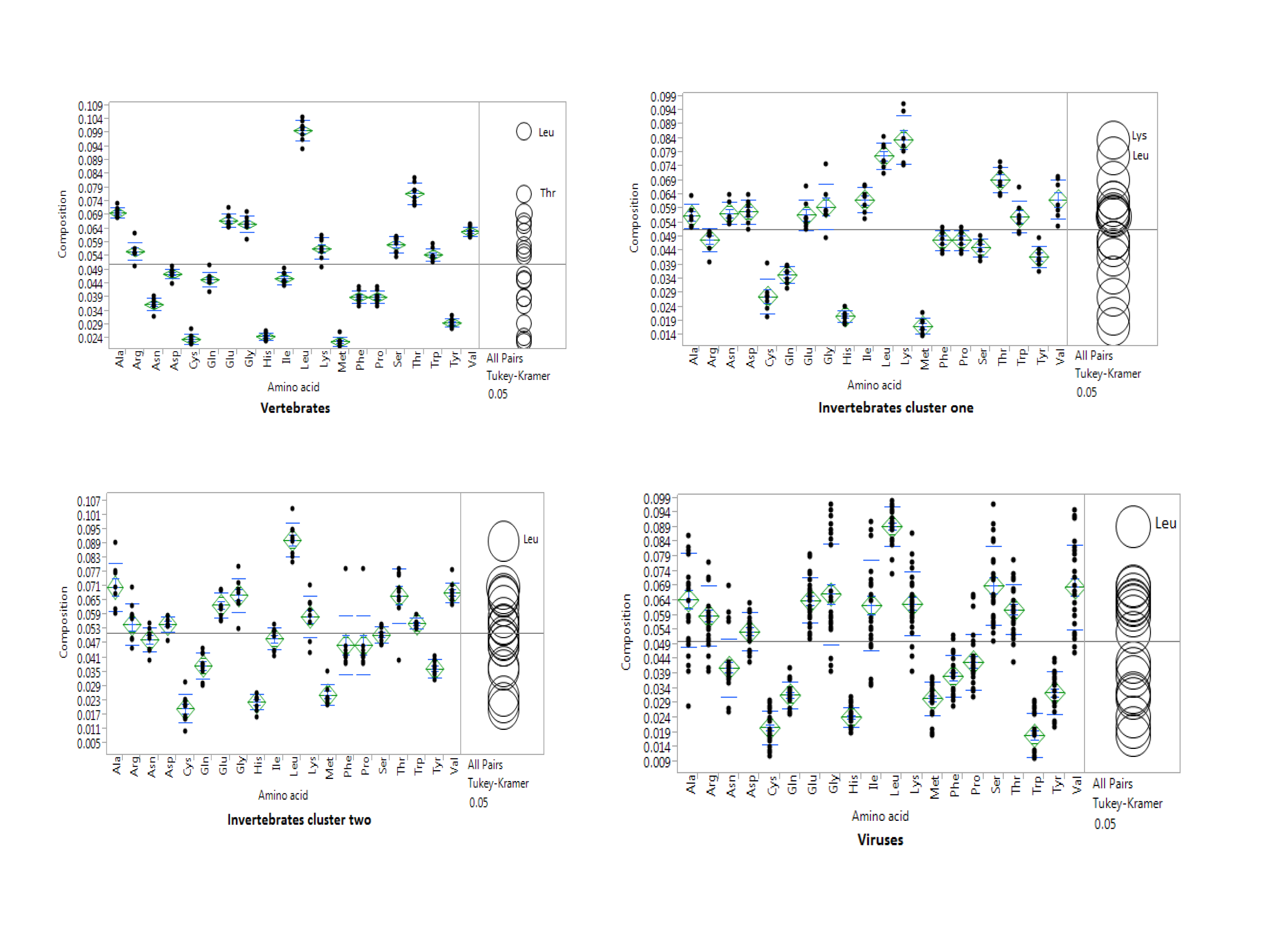

Supplement: S1 Fig — Tukey-Kramer analysis conducted among vertebrate, invertebrate and virus groups to determinate the frequency of amino acids among the proteins analyzed in this study. (TIF) [file pone.0159943.s001.tif]
